# Could Sleep Be an Antidote to Optic Disc Edema in Astronauts?

**DOI:** 10.3390/life15020183

**Published:** 2025-01-26

**Authors:** Peter Wostyn, Maiken Nedergaard

**Affiliations:** 1 Department of Psychiatry, PC Sint-Amandus, 8730 Beernem, Belgium; 2 Center for Translational Neuromedicine, Faculty of Health and Medical Sciences, University of Copenhagen, 2200 Copenhagen, Denmark; nedergaard@sund.ku.dk; 3 Center for Translational Neuromedicine, University of Rochester Medical Center, Rochester, NY 14642, USA

**Keywords:** astronaut, ocular glymphatic system, optic disc edema, sleep, spaceflight associated neuro-ocular syndrome

## Abstract

A spectrum of neuro-ocular changes has been observed in astronauts during and after prolonged exposure to microgravity on long-duration spaceflights. These changes, collectively referred to as “spaceflight associated neuro-ocular syndrome” (SANS), pose a significant challenge for space agencies as they prepare for future human missions, including a return to the Moon and manned missions to Mars. Optic disc edema, a hallmark feature of SANS, occurs in approximately 70% of astronauts on extended missions. Recent evidence suggests a potential link between poor sleep and the development of optic disc edema in individuals exposed to a spaceflight analog environment, providing critical insights into its underlying pathophysiology. Here, we propose a novel hypothesis: sleep deprivation may increase the risk of microgravity-induced optic disc edema by altering translaminar pressure dynamics and disrupting ocular glymphatic outflow. This perspective offers a new framework for understanding SANS and highlights potential targets to mitigate its risks in the context of human space exploration.

## 1. Introduction

**Figure 1:**
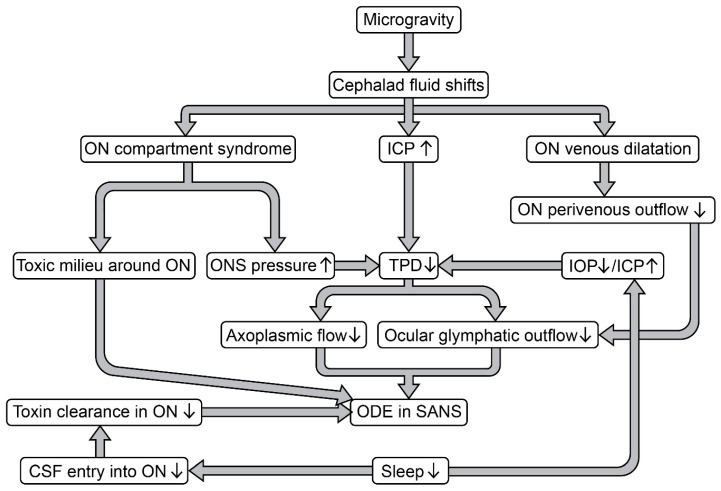
Optic disc edema in spaceflight associated neuro-ocular syndrome: previously proposed hypotheses relevant to the two alternative hypotheses. Flowchart illustrating previously proposed hypotheses for spaceflight associated neuro-ocular syndrome (SANS) in relation to the two alternative hypotheses explaining the link between impaired sleep and optic disc edema (ODE) in subjects exposed to a spaceflight analog. The first hypothesis concerns the “retrograde ocular glymphatic cerebrospinal fluid (CSF) pathway”. According to this hypothesis, reduced sleep may contribute to ODE development by disrupting the retrograde ocular glymphatic system, impairing metabolic waste clearance from the optic nerve (ON) region. The second hypothesis involves the “anterograde ocular glymphatic clearance pathway”. This hypothesis posits that sleep deprivation may increase the risk of ODE by altering translaminar pressure dynamics and disrupting ocular glymphatic outflow. ICP, intracranial pressure; IOP, intraocular pressure; ONS, optic nerve sheath; TPD, translaminar pressure difference.

Ophthalmic changes, including optic disc edema (ODE), globe flattening, chorioretinal folds, and hyperopic refractive error shifts, have been consistently observed in astronauts during and after long-duration spaceflight [1,2,3]. Collectively, these neuro-ocular findings are referred to as “spaceflight associated neuro-ocular syndrome” (SANS) [2,3]. Understanding the factors contributing to SANS has become a top priority for space agencies, particularly in the context of planned human missions to the Moon and Mars [2,3]. ODE, a hallmark feature of SANS, has been reported in approximately 70% of astronauts completing extended missions aboard the International Space Station, as quantified by optical coherence tomography [4]. While the exact mechanisms underlying SANS-associated ODE remain unclear, chronic headward fluid shifts in microgravity are hypothesized to contribute to mildly elevated intracranial pressure (ICP) [1,3]. Lawley et al. [5] speculated that microgravity prevents the normal reduction in ICP that occurs in the upright posture on Earth, resulting in a reduced translaminar pressure difference, defined as the difference between intraocular pressure (IOP) and ICP (Figure 1). Pathologically elevated ICP, resulting in stasis of axoplasmic flow and axonal swelling, was initially proposed as a potential mechanism for the development of ODE in SANS (Figure 1) [1]. However, emerging evidence indicates that SANS-associated ODE does not mirror terrestrial idiopathic intracranial hypertension (IIH) [1,3]. Postmission lumbar punctures in astronauts reveal only borderline elevated ICPs, and typical symptoms of terrestrial IIH, such as severe headache, pulsatile tinnitus, or diplopia, have not been reported by astronauts [1,3]. An alternative hypothesis suggests that ODE in astronauts results from localized cerebrospinal fluid (CSF) compartmentalization within the orbital optic nerve sheath, with locally elevated CSF pressures rather than solely increased ICP (Figure 1) [1]. This compartmentalization may result from disrupted flow equilibrium in the tightly confined and septated orbital subarachnoid space, exacerbated by prolonged headward fluid shifts during microgravity exposure [1].

Emerging evidence linking poor sleep to the development of ODE in subjects exposed to a spaceflight analog environment provides new insights into the pathophysiology of ODE in SANS [6]. Sleep deficiency is a well-documented challenge among crewmembers during both short- and long-duration spaceflights [4]. However, no studies to date have directly examined whether reduced sleep durations in space increase the risk of developing ODE. Building on the concept of the “anterograde ocular glymphatic system”, we propose a novel hypothesis: sleep deprivation may increase the risk of ODE in SANS by altering translaminar pressure dynamics and disrupting ocular glymphatic outflow (Figure 1). This perspective highlights the need for further research to explore the interplay between sleep, microgravity, and ocular health, advancing our understanding of SANS and informing strategies to mitigate its risks during future space exploration.

## 2. Discussion

### 2.1. Sleep and Optic Disc Edema in a Spaceflight Analog

Christian et al. [6] studied the effects of 30 days of 6° head-down tilt bed rest (HDTBR) combined with mildly elevated carbon dioxide levels in 11 healthy subjects (five women). This spaceflight analog study found that individuals who developed ODE (*n* = 5) experienced shorter sleep durations, reduced non-rapid eye movement (non-REM) sleep stage 2, increased wake after sleep onset, and a blunted circadian temperature amplitude. Given the activation of the brain glymphatic system during sleep [7], the authors hypothesized that reduced sleep impairs glymphatic clearance in the ocular system, leading to metabolic waste accumulation at the optic nerve head [6]. They speculated that, when combined with chronic cephalad fluid shifts, this impairment could predispose individuals to ODE (Figure 1) [6].

Building on these findings, we propose a complementary hypothesis involving the “anterograde ocular glymphatic system” to explain the link between sleep deprivation and the development of ODE in a spaceflight analog environment. We first explore the role of the “retrograde ocular glymphatic CSF pathway” in this association.

### 2.2. Sleep and Optic Disc Edema in SANS: A Retrograde Ocular Glymphatic Perspective

**Figure 2:**
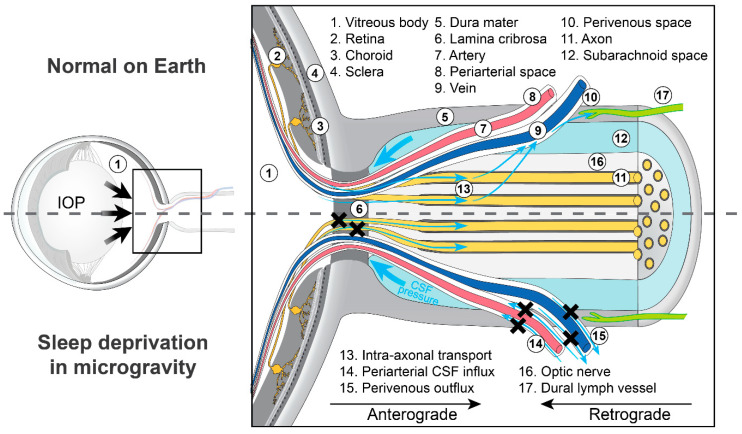
Sleep and optic disc edema in spaceflight associated neuro-ocular syndrome from an ocular glymphatic perspective. The figure illustrates the influence of sleep deprivation on ocular glymphatic transport under microgravity conditions compared to normal ocular glymphatic function on Earth. Top half of the figure: In a healthy eye on Earth, intraocular fluid and solutes, such as amyloid-β, are transported intra-axonally along the retinal ganglion cell axons across the lamina barrier. Upon passing the lamina region, these fluids and solutes are released and accumulate along perivenous spaces in the optic nerve prior to entering dural lymphatics around the nerve. The bottom half of the figure illustrates sleep- and microgravity-related changes to the ocular glymphatic system in the early stage of optic disc edema. Two potential mechanisms link sleep deprivation to optic disc edema. According to the first hypothesis, impaired sleep in microgravity may result in decreased glymphatic clearance due to reduced cerebrospinal fluid (CSF) entry into the optic nerve. This leads to the build-up of metabolic waste at the optic nerve head. Coupled with chronic cephalad fluid shifts in microgravity, this may increase the risk of optic disc edema. According to the alternative hypothesis, sleep deprivation in microgravity may lower the translaminar pressure difference (caused by reduced intraocular pressure (IOP) and elevated intracranial pressure). This pressure imbalance reduces ocular glymphatic outflow, leading to fluid stasis within the prelaminar optic nerve head. Microgravity-induced cephalad fluid shifts exacerbate these effects by further increasing orbital CSF pressure and reducing translaminar pressure difference. Additionally, compromised perivenous outflow in the optic nerve (due to closure of perivenous spaces) worsens ocular glymphatic outflow, thereby increasing the risk of optic disc edema development.

**Figure 3:**
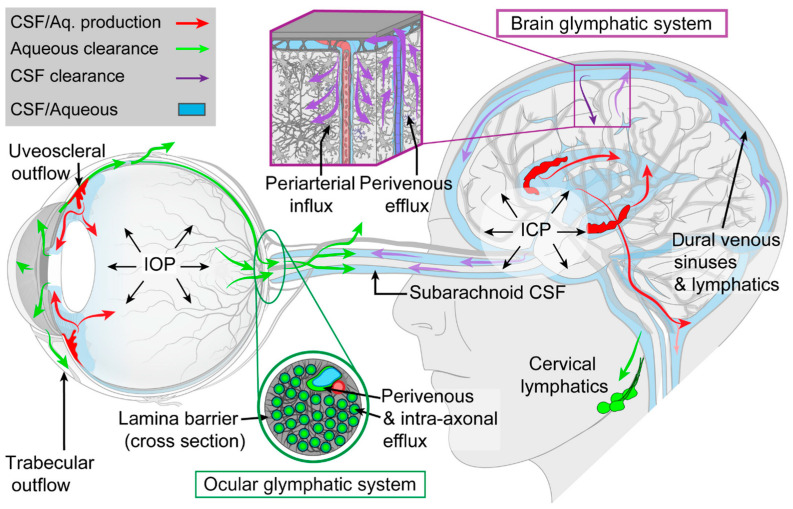
The glymphatic systems in the brain and the eye. This macroscopic overview illustrates the glymphatic systems in the brain and the eye, emphasizing the roles of pressure gradients, hydrostatic barriers, and lymphatic drainage, shown in the context of known outflow pathways for cerebrospinal fluid (CSF) and aqueous humor. In the brain, CSF flows from subarachnoid spaces into periarterial spaces, where it exchanges with interstitial fluid within the parenchyma. Interstitial solutes, such as amyloid-β, are then cleared from the brain along perivenous spaces. These solutes are ultimately drained via meningeal lymphatic vessels into cervical lymph nodes. The anterograde ocular glymphatic clearance pathway enables fluid and waste removal from the posterior eye into the optic nerve. Intraocularly administered tracers like amyloid-β enter retinal ganglion cell axons and the perivenous spaces of the retina and optic nerve before being cleared by dural lymphatics surrounding the optic nerve, and eventually, cervical lymph nodes. ICP, intracranial pressure; IOP, intraocular pressure. Figure reproduced from Rangroo Thrane et al. [9] Creative Commons.

The first hypothesis suggests that impaired sleep may reduce glymphatic clearance along the “retrograde ocular glymphatic CSF pathway” (directed from the brain toward the eye), resulting in metabolic waste accumulation and an increased risk of ODE in SANS (Figure 1 and Figure 2) [6]. In the brain, glymphatic transport involves CSF entering periarterial spaces, mixing with interstitial fluid, and clearing solutes, including amyloid-β (Aβ), via perivenous spaces and lymphatic drainage pathways (Figure 3) [8,9]. This process is facilitated by aquaporin-4 (AQP4) water channels, which are expressed in a highly polarized manner on the astrocytic endfeet ensheathing the cerebral vasculature [8]. A key finding is that the brain glymphatic system is suppressed during wakefulness but is predominantly active during sleep, particularly during non-REM slow-wave sleep [7]. Experimental studies in mice demonstrated a several-fold increase in glymphatic CSF influx and a doubling of Aβ clearance during sleep compared to wakefulness [7,10]. Furthermore, a 60% expansion of interstitial volume during sleep was observed, which reduces resistance to fluid flow [7].

In 2015, Wostyn et al. [11] proposed the possibility of a similar glymphatic pathway existing in the optic nerve. In a subsequent post-mortem study, the authors examined cross-sections of human optic nerves using light microscopy after injecting India ink into the optic nerve’s subarachnoid space [12]. They observed a striking accumulation of India ink in perivascular spaces, supporting the presence of an ocular glymphatic pathway or at least a perivascular system [12]. Since then, several studies have confirmed glymphatic-like fluid transport from the brain to the optic nerve [13,14,15]. In live mice, Mathieu et al. [13] demonstrated that CSF tracers injected into the cisterna magna travel along the optic nerve, specifically within spaces immediately surrounding blood vessels. Furthermore, they showed that these CSF tracer channels in the optic nerve are bordered by AQP4-positive astrocytic endfeet [13]. These findings were confirmed and extended in a more recent study by Wang et al. [14] in mice, which showed that tracers injected into the cisterna magna predominantly enter the optic nerve via periarterial spaces. These tracers then exit through dural lymphatics surrounding the optic nerve and cervical lymphatic vessels, providing additional evidence of glymphatic clearance mechanisms in visual structures [16]. Lymphatics in the dura mater of the human optic nerve were initially described by Killer et al. [17] and Gausas et al. [18] in 1999. The existence of a glymphatic pathway in visual structures has also been suggested in humans using magnetic resonance imaging [15]. Following intrathecal administration of gadobutrol as a CSF tracer, Jacobsen et al. [15] noted tracer enrichment within the visual pathway, from the primary visual cortex over the optic tract and optic chiasm as well as the optic nerve. Notably, substantial CSF tracer enrichment was observed in the retrobulbar segment of the optic nerve compared to the middle and posterior parts, corresponding to the entry of the central retinal artery [15]. The authors hypothesized that this area serves as a major periarterial pathway, facilitating CSF tracer entry from the subarachnoid space into the optic nerve interstitium, similar to the primary routes of CSF tracer entry into the human brain parenchyma along major cerebral arteries [15].

Given that the optic nerve is an extension of the diencephalon [19] and, developmentally, is part of the brain, and given that the brain glymphatic system is predominantly active during sleep [7], impaired sleep was speculated to contribute to decreased glymphatic clearance in the optic nerve, potentially increasing the risk of developing microgravity-induced ODE (Figure 1 and Figure 2) [4,6]. In line with this hypothesis, metabolic toxicity, more specifically, the sequestration of CSF in the subarachnoid space of the optic nerve, leading to a toxic milieu around the nerve, has been previously proposed as a possible mechanism for terrestrial papilledema and ODE in astronauts (Figure 1) [20,21]. However, an important unresolved question is whether the retrograde ocular glymphatic transport of CSF is sleep-dependent, as has been observed for the brain’s glymphatic system [7]. To date, no studies have directly examined the impact of sleep deprivation on ocular glymphatic function. This gap in understanding is critical as these findings collectively suggest that glymphatic dysfunction, exacerbated by sleep deprivation and cephalad fluid shifts in microgravity, may underlie ODE in SANS. Further research is essential to clarify the interplay between sleep, glymphatic dynamics, and ocular health in astronauts. Such investigations could provide crucial insights, paving the way for targeted interventions to mitigate the risks associated with SANS during extended space missions.

### 2.3. An Alternative Hypothesis: Sleep and Optic Disc Edema in SANS from an Anterograde Ocular Glymphatic Perspective

#### 2.3.1. The Anterograde Ocular Glymphatic Pathway and Its Role in SANS

Here, we propose an alternative hypothesis for the role of sleep in the development of ODE in SANS, focusing on the “anterograde ocular glymphatic clearance pathway” (Figure 1, Figure 2 and Figure 3). In 2017, Wostyn et al. [22] proposed that ODE in astronauts may result, at least in part, from a glymphatic flow imbalance at the optic nerve head. This imbalance, linked to a reduced translaminar pressure difference, was hypothesized to cause prelaminar fluid accumulation (Figure 1) [22]. Supporting this hypothesis, research conducted by the Nedergaard laboratory in 2020 identified an “anterograde ocular glymphatic clearance system” in rodents (Figure 3) [14]. Their findings demonstrated the transport of intravitreally injected tracers, such as Aβ, through an intra-axonal route in retinal ganglion cell axons and via perivenous spaces in the retina and optic nerve head [9,14]. Once tracers crossed the lamina barrier, they were transported through perivenous spaces in the optic nerve, exiting via dural lymphatics surrounding the optic nerve, and eventually draining into cervical lymph nodes (Figure 2) [14].

Unlike the brain’s glymphatic clearance, which is predominantly regulated by sleep [7], the anterograde glymphatic pathway in the eye is driven by the translaminar pressure difference and light-induced pupil constriction [14]. In healthy eyes, the posteriorly directed translaminar pressure gradient facilitates effective glymphatic outflow from the eye. Although rodent models lack the collagenous lamina cribrosa present in humans, these findings provide a critical foundation for further exploration of ocular glymphatic mechanisms in humans. If confirmed in humans, increased orbital CSF pressure in microgravity could hinder glymphatic efflux by reducing the translaminar pressure difference, resulting in fluid accumulation at the optic nerve head, potentially contributing to the development of ODE in SANS (Figure 1 and Figure 2) [22].

#### 2.3.2. The Role of Sleep in Translaminar Pressure Dynamics and Optic Disc Edema in SANS

Sleep significantly influences translaminar pressure dynamics. Studies in healthy subjects have shown that IOP is lower during wakefulness than during REM and non-REM sleep stages [23]. Similarly, a study investigating the effect of prolonged simulated microgravity (6° HDT) on ICP reported that, compared to the awake supine posture, ICP decreased by ~2–4 mmHg during sleep and returned to the supine baseline value after 24 h of 6° HDT [5]. If studies confirm these findings in microgravity, the lower translaminar pressure difference during wakefulness may create a dysregulation of the fine translaminar pressure balance, potentially reducing ocular glymphatic outflow and leading to fluid stasis within the prelaminar optic nerve head (Figure 1 and Figure 2). This mechanism could explain the reported link between impaired sleep and ODE in subjects exposed to a spaceflight analog [6]. To explore and validate this hypothesis, it would be necessary to develop a non-invasive monitoring system capable of continuously measuring IOP and ICP in astronauts during spaceflight or under analog conditions. In microgravity, cephalad fluid shifts may further lower the translaminar pressure difference due to an increase in orbital CSF pressure. Additionally, these headward fluid shifts may impair venous drainage from the eye, potentially collapsing perivenous spaces. Both effects could exacerbate reductions in ocular glymphatic outflow (Figure 1 and Figure 2). Combined with increased capillary filtration in the prelaminar optic nerve head which lacks classical blood-brain barrier characteristics [24], fluid transudation from the choroidal vasculature [25], and the entry of excess CSF substrate into the eye [22,26], these factors may collectively lead to prelaminar fluid overload and ODE development. These mechanisms, especially under the unique conditions of microgravity, warrant further investigation to mitigate the risks associated with SANS during long-duration spaceflight.

## 3. Conclusions

This Perspective underscores the association between impaired sleep and the development of ODE in a spaceflight analog environment. We propose that sleep deprivation may increase the risk of microgravity-induced ODE by disrupting translaminar pressure dynamics and ocular glymphatic outflow. Validating this hypothesis necessitates the development of non-invasive monitoring systems capable of continuously measuring IOP and ICP in astronauts during spaceflights or under analog conditions. Further research into the interplay between sleep, glymphatic dynamics, and microgravity is crucial for elucidating the mechanisms underlying ODE in SANS and for mitigating its risks during long-duration space missions.

## References

[1] Mader T.H., Gibson C.R., Pass A.F., Kramer L.A., Lee A.G., Fogarty J., Tarver W.J., Dervay J.P., Hamilton D.R., Sargsyan A. (2011). Optic disc edema, globe flattening, choroidal folds, and hyperopic shifts observed in astronauts after long-duration space flight. Ophthalmology.

[2] Lee A.G., Mader T.H., Gibson C.R., Brunstetter T.J., Tarver W.J. (2018). Space flight-associated neuro-ocular syndrome (SANS). Eye.

[3] Lee A.G., Mader T.H., Gibson C.R., Tarver W. (2017). Space flight-associated neuro-ocular syndrome. JAMA Ophthalmol..

[4] Nguyen T., Ong J., Waisberg E., Lee A.G. (2024). Sleep and optic disc edema in spaceflight associated neuro-ocular syndrome (SANS). Eye.

[5] Lawley J.S., Petersen L.G., Howden E.J., Sarma S., Cornwell W.K., Zhang R., Whitworth L.A., Williams M.A., Levine B.D. (2017). Effect of gravity and microgravity on intracranial pressure. J. Physiol..

[6] Christian K.H., Petitti C., Oretga-Schwartz K., Mulder E., Noppe A., von der Wiesche M., Stern C., Young M., Macias B.R., Laurie S.S. (2024). Development of optic disc edema during 30 days of hypercapnic head-down tilt bed rest is associated with short sleep duration and blunted temperature amplitude. J. Appl. Physiol..

[7] Xie L., Kang H., Xu Q., Chen M.J., Liao Y., Thiyagarajan M., O’Donnell J., Christensen D.J., Nicholson C., Iliff J.J. (2013). Sleep drives metabolite clearance from the adult brain. Science.

[8] Iliff J.J., Wang M., Liao Y., Plogg B.A., Peng W., Gundersen G.A., Benveniste H., Vates G.E., Deane R., Goldman S.A. (2012). A paravascular pathway facilitates CSF flow through the brain parenchyma and the clearance of interstitial solutes, including amyloid β. Sci. Transl. Med..

[9] Rangroo Thrane V., Hynnekleiv L., Wang X., Thrane A.S., Krohn J., Nedergaard M. (2021). Twists and turns of ocular glymphatic clearance—New study reveals surprising findings in glaucoma. Acta Ophthalmol..

[10] Tithof J., Boster K.A.S., Bork P.A.R., Nedergaard M., Thomas J.H., Kelley D.H. (2022). A network model of glymphatic flow under different experimentally-motivated parametric scenarios. iScience.

[11] Wostyn P., Van Dam D., Audenaert K., Killer H.E., De Deyn P.P., De Groot V. (2015). A new glaucoma hypothesis: A role of glymphatic system dysfunction. Fluids Barriers CNS.

[12] Wostyn P., De Groot V., Van Dam D., Audenaert K., De Deyn P.P., Killer H.E. (2016). The glymphatic system: A new player in ocular diseases?. Investig. Ophthalmol. Vis. Sci..

[13] Mathieu E., Gupta N., Ahari A., Zhou X., Hanna J., Yücel Y.H. (2017). Evidence for cerebrospinal fluid entry into the optic nerve via a glymphatic pathway. Investig. Ophthalmol. Vis. Sci..

[14] Wang X., Lou N., Eberhardt A., Yang Y., Kusk P., Xu Q., Förstera B., Peng S., Shi M., Ladrón-de-Guevara A. (2020). An ocular glymphatic clearance system removes β-amyloid from the rodent eye. Sci. Transl. Med..

[15] Jacobsen H.H., Ringstad G., Jørstad Ø.K., Moe M.C., Sandell T., Eide P.K. (2019). The human visual pathway communicates directly with the subarachnoid space. Investig. Ophthalmol. Vis. Sci..

[16] Delle C., Wang X., Nedergaard M. (2024). The ocular glymphatic system-current understanding and future perspectives. Int. J. Mol. Sci..

[17] Killer H.E., Laeng H.R., Groscurth P. (1999). Lymphatic capillaries in the meninges of the human optic nerve. J. Neuroophthalmol..

[18] Gausas R.E., Gonnering R.S., Lemke B.N., Dortzbach R.K., Sherman D.D. (1999). Identification of human orbital lymphatics. Ophthalmic Plast. Reconstr. Surg..

[19] Kasi A., Liu C., Faiq M.A., Chan K.C. (2022). Glymphatic imaging and modulation of the optic nerve. Neural Regen. Res..

[20] Killer H.E., Jaggi G.P., Miller N.R. (2009). Papilledema revisited: Is its pathophysiology really understood?. Clin. Exp. Ophthalmol..

[21] Mader T.H., Gibson C.R., Otto C.A., Sargsyan A.E., Miller N.R., Subramanian P.S., Hart S.F., Lipsky W., Patel N.B., Lee A.G. (2017). Persistent asymmetric optic disc swelling after long-duration space flight: Implications for pathogenesis. J. Neuroophthalmol..

[22] Wostyn P., Killer H.E., De Deyn P.P. (2017). Why a one-way ticket to Mars may result in a one-way directional glymphatic flow to the eye. J. Neuroophthalmol..

[23] Aptel F., Canaud P., Tamisier R., Pépin J.L., Mottet B., Hubanova R., Romanet J.-P., Chiquet C. (2015). Relationship between nocturnal intraocular pressure variations and sleep macrostructure. Investig. Ophthalmol. Vis. Sci..

[24] Macias B.R., Patel N.B., Gibson C.R., Samuels B.C., Laurie S.S., Otto C., Ferguson C.R., Lee S.M.C., Ploutz-Snyder R., Kramer L.A. (2020). Association of long-duration spaceflight with anterior and posterior ocular structure changes in astronauts and their recovery. JAMA Ophthalmol..

[25] Wostyn P., Gibson C.R., Mader T.H. (2022). Optic disc edema in astronauts from a choroidal point of view. Aerosp. Med. Hum. Perform..

[26] Wostyn P., Mader T.H., Gibson C.R., Nedergaard M. (2025). New insights in brain-to-eye transport: Can excess cerebrospinal fluid in astronauts escape into the eye?. Eye.

